# The Lateral Approach in the Surgical Treatment of a Complex Dorsal Metacarpophalangeal Joint Dislocation of the Index Finger

**DOI:** 10.1155/2019/1063829

**Published:** 2019-04-10

**Authors:** Joana Monteiro Pereira, Miguel Quesado, Marcos Silva, João Das Dores Carvalho, Hélder Nogueira, Jorge Alves

**Affiliations:** Centro Hospitalar Tâmega e Sousa, Penafiel, Portugal

## Abstract

Complex dorsal metacarpophalangeal (MCP) joint dislocations as a result of hyperextension injuries are uncommon in the pediatric population and irreducible to closed maneuvers. Treatment of these complex lesions is invariably surgical, and dorsal or volar approaches are traditionally used. The authors describe a case of a 16-year-old male who suffered a fall onto his outstretched right hand in a soccer game. The patient presented to the ER with pain and deformity of the index finger MCP joint. Radiographs confirmed a complex MCP dislocation with a small osteochondral fragment. A lateral surgical approach was made, and interposition of the volar plate and an osteochondral fragment blocking the reduction were found. This versatile approach allowed access to volar and dorsal structures, minimizing the risk of surgical scarring and mobility arch limitation. To our knowledge, there are no reported cases regarding a lateral surgical approach.

## 1. Introduction

Traumatic hand injuries are common in pediatric sports [1]. Most cases present as interphalangeal joint sprains or phalangeal fractures [2].

With the exception of the thumb, metacarpophalangeal (MCP) joints are protected by their anatomical location and strong ligament complexes [3]. MCP joint dislocation is a rare entity, even in the pediatric population. The peripheral position of the index and small fingers makes them more susceptible to this kind of injury [4].

Dislocations can be classified as simple (more frequent) or complex if an associated fracture or soft tissue interposition prevents closed reduction.

The pathogenesis of irreducible dislocations refers to cases where the volar plate is avulsed from its own attachment to the metacarpal and becomes interposed between the proximal phalanx and the metacarpal. The presence of osteochondral fragments may require fixation or excision [3].

The longer the dislocation remains unreduced, the more likely complications such as loss of motion, degenerative arthritis, and osteonecrosis will occur. In skeletally immature patients with this injury, the surgeon must be mindful that premature closure of the physis and metacarpal shortening can occur [3].

Treatment of complex lesions is surgical and can be done by a dorsal approach, a volar approach, or a combined one [5].

The volar approach allows better volar plate visualization, but successful reduction cannot always be obtained through this approach, and the neurovascular bundle can easily be damaged. More recent evidence suggests that a dorsal approach allows an easier MCP joint reduction than would the volar, objectively defined as a decreased operative time.

The best surgical approach to treat this problem is not consensual among surgeons [6].

The authors present a rare case of an index finger MCP dislocation surgically treated by a new MCP lateral approach that prevents soft tissue disruption, allows a quick and good reduction, and decreases the risk of subsequent stiffness.

## 2. Case Report

To our knowledge, this is the first reported case of an index finger MCP joint dislocation surgically treated by a lateral approach.

**Figure 1:**
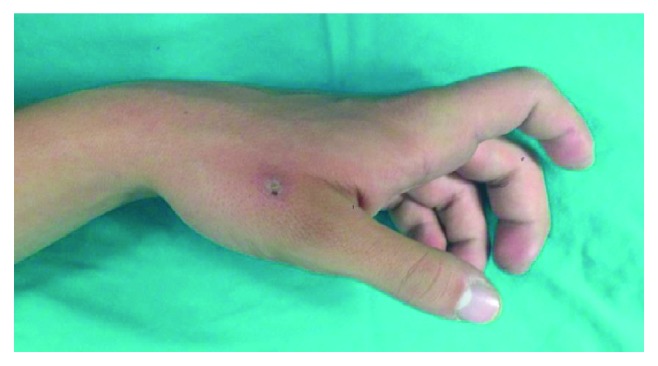
Deformity in hyperextension of the MCP index joint with prominence of the 2nd metacarpal head.

The authors describe a case of a 16-year-old male who suffered a fall onto his outstretched right hand during a soccer game. The patient presented to the ER with pain and deformity of the index finger MCP joint. Volarly, the prominence of the second metacarpal head was evident (Figure 1).

**Figure 2:**
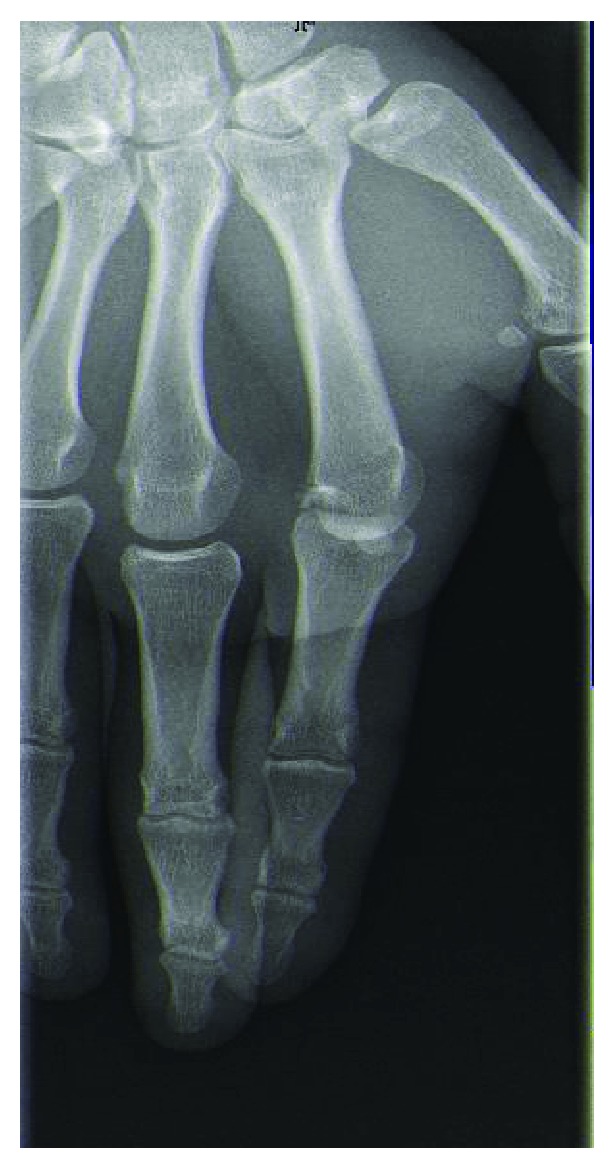
X-Ray (AP view) showing MCP dislocation of the index finger.

**Figure 3:**
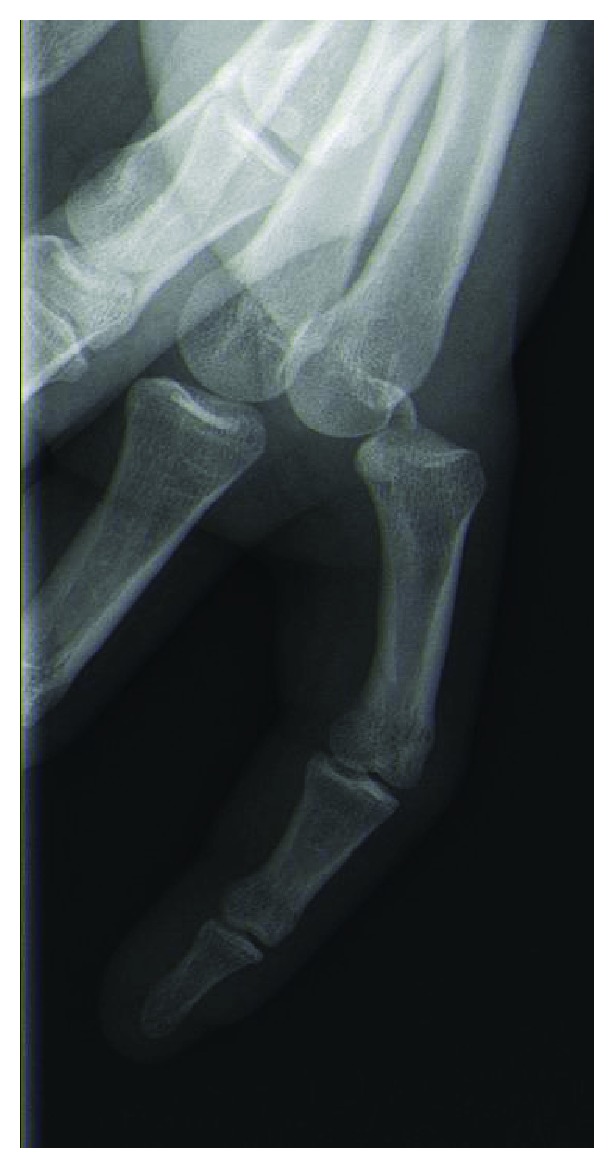
X-Ray (lateral view) showing MCP dislocation of the index finger with a small dorsal osteochondral fragment.

Radiographs confirmed a dorsal index finger MCP joint dislocation and showed a small dorsal osteochondral fragment (Figures 2 and 3).

After multiple unsuccessful reduction attempts under ring block by different physicians, the patient was referred to surgery.

**Figure 4:**
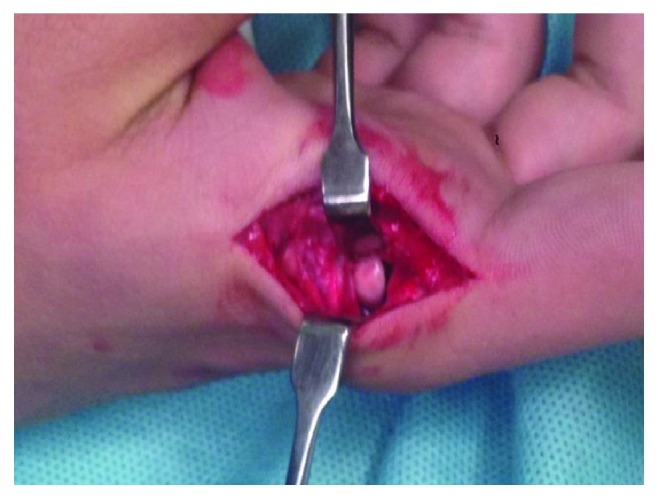
Lateral approach on the MCP joint.

Under general anesthesia, a lateral surgical approach (Figure 4) was performed on the MCP joint. A straight longitudinal incision was made over the lateral aspect of the MCP joint; the volar neurovascular bundle and the dorsal branch of the digital nerve were identified and retracted with *Farabeufs*.

**Figure 5:**
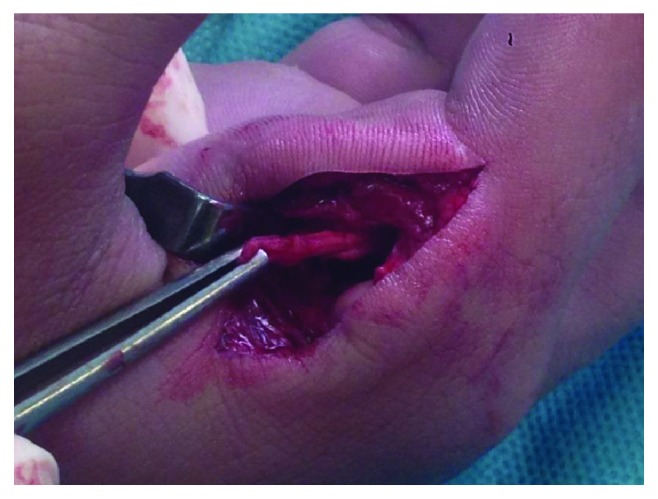
Interposition of the volar plate blocking the reduction.

Interposition of the volar plate (Figure 5) preventing the reduction was observed. Applying gentle traction and flexion, the MCP joint was reduced, and proximal volar plate reinsertion with a 4-0 Vicryl suture was performed.

**Figure 6:**
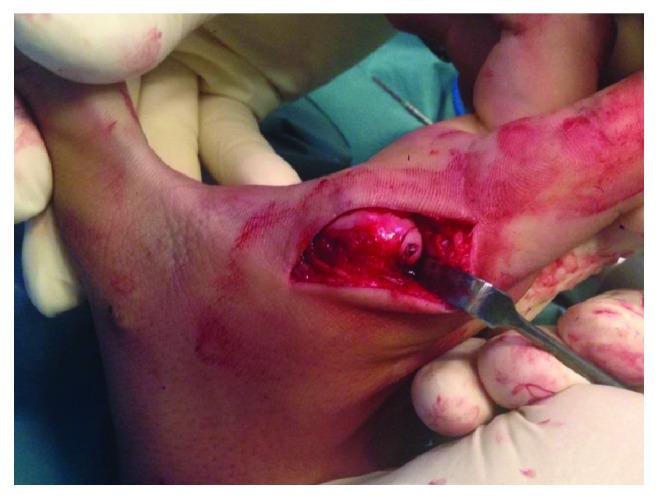
Fixation of the dorsal osteochondral fragment.

The posterior joint capsule was identified and split longitudinally, above the collateral ligament. Once adequately exposed, a small osteochondral fragment was found (Figure 6). Reduction and retrograde fixation of the osteochondral fragment with a 1.7 mm screw were performed, burying the screw head in the cartilage.

The joint capsule, subcutaneous layer, and skin were closed using appropriate sutures. Reduction was confirmed by intraoperative fluoroscopy.

The patient was placed in a volar splint with approximately 45° of flexion and discharged on postoperative day zero without any complications.

Immobilization was removed by week 3. Radiographic control revealed joint congruence, and the patient was encouraged to actively mobilize the finger.

**Figure 7:**
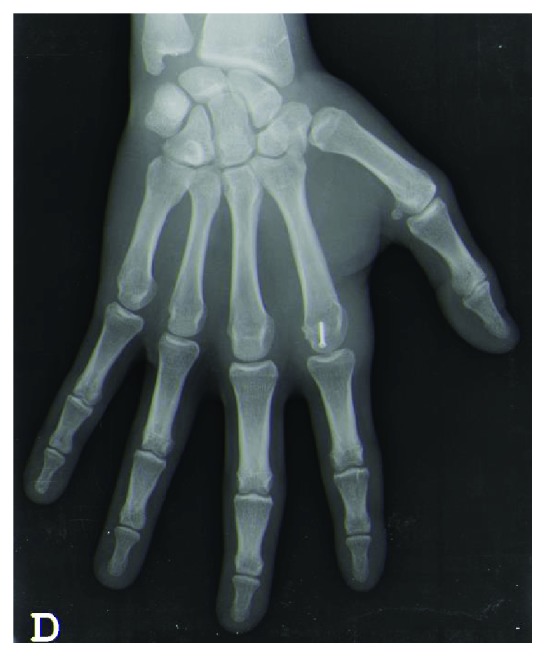
X-Ray (AP view) showing fracture consolidation at week 6.

**Figure 8:**
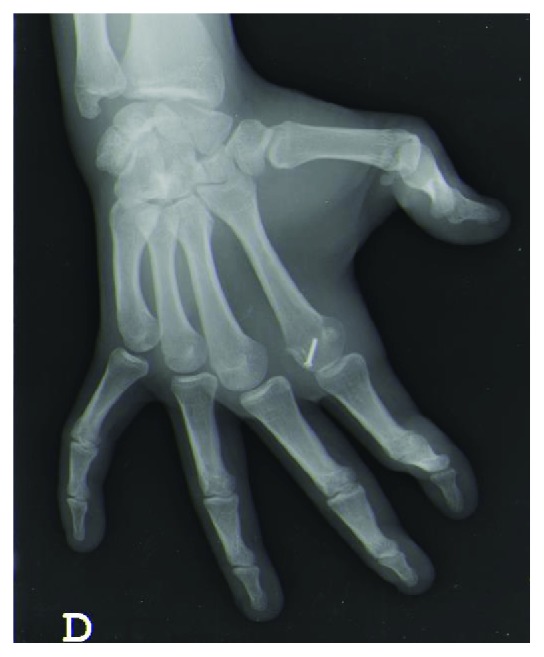
X-Ray (lateral view) showing fracture consolidation at week 6.

At week 6, the fracture was consolidated (Figures 7 and 8). The joint was painless and presented slight stiffness (ROM 0-70°). The patient could return to competition with protective syndactyly.

One year postoperative, there was no pain, growth disturbance, or joint stiffness, with full ROM of the index finger.

## 3. Discussion

MCP joint dislocations are relatively uncommon and occur less often than interphalangeal joint dislocations.

Complex pediatric MCP joint dislocations occur in a similar fashion as those in adults, most commonly in the index and little fingers. This kind of injury requires a surgical approach for reduction and proper alignment [7].

The MCP joint, in addition to the collateral ligaments, is reinforced by the volar plate and transverse palmar ligament. Hyperextension can lead to rupture of the volar structures. If the movement is continued, the volar plate might become positioned dorsally to the metacarpal head, blocking the reduction. In complex dorsal MCP joint dislocations, the volar plate has been identified as the most significant barrier to reduction [8].

Volar MCP joint dislocations are less common than dorsal dislocations, and different structures are involved in complex lesions (dorsal capsule, distal insertion of the volar plate, and the tendinous junction) [8].

In surgical management, dorsal or volar approaches are traditionally used [6]. Farabeuf first described the dorsal approach in 1876 [9] while Kaplan described the volar approach in 1957 [10].

Volar or dorsal approaches are both viable options in the treatment of complex MCP dislocations. Each approach has its own advantages and disadvantages, and controversy remains about which one is superior.

The dorsal approach may offer a critical advantage in decreasing risk of neurovascular injury, as well as the ability to manage associated osteochondral fractures [11]. This approach is recommended for the infrequent hand surgeon as a safe choice with stable results [8].

The volar approach is recommended for experienced hand surgeons as it allows for a complete anatomic restoration of the joint to be achieved and repair of the volar plate, which may decrease the risk of late instability [12].

An MCP joint dislocation displaces the neurovascular bundle superficially and immediately under the skin, placing it at risk in the volar approach [2].

In addition, less invasive techniques performed on pediatric patients have been described: arthroscopic surgery or percutaneous techniques. Although information is limited, the percutaneous techniques may be worth considering in complex MCP joint dislocations [13].

The new lateral surgical approach has a risk of nerve and vessel injuries. The risk is low, but the injury is severe and therefore to avoid. A careful preservation of the volar neurovascular bundle and dorsal branches of the digital nerve with a *Farabeuf* retractor prevents the risk of lesion.

The special advantage of this new technique is the visualization and treatment of both volar and dorsal structures: reinsertion of the volar plate, as well as an easy access to fixation of the osteochondral fragment of the dorsal portion of the metacarpal head.

The authors believe that the operative scar in the lateral approach may reduce the risk of tendon adhesions, as well as scar retractions that may limit joint movement (a common complication). In this case, the patient had a normal motion and function at the end of the follow-up period, with no hand disability, premature epiphysis closure, or metacarpal shortening.

## 4. Conclusion

In this clinical case, it is important to highlight the rarity of the lesion in pediatric athletes.

Complex MCP dislocations with an interposed osteochondral fragment should be approached surgically. In this particular case, the need for anatomical reduction of the fragment and its rigid fixation must be emphasized, being careful to bury the screw head in the cartilage.

Urgent treatment mostly leads to good prognosis with an early return to sports activity. Joint stiffness is the most common complication possibly resulting from soft tissue trauma at the time of injury, from prolonged immobilization, or from osteochondral fracture and related degenerative changes [2].

In conclusion, dorsal and volar approaches are the most common surgical techniques used to reduce complex MCP dislocations, although controversy exists regarding which one is preferable.

The lateral approach seems a good alternative. It is a versatile approach that allows access to both volar and dorsal structures and probably minimizes the risk of complications with postoperative scarring.

To our knowledge, there are no reported cases regarding a lateral surgical approach.
